# Phasor-based FLIM analysis of NAD(P)H and FAD autofluorescence for label-free bacterial classification

**DOI:** 10.1117/1.JBO.31.1.016502

**Published:** 2026-01-17

**Authors:** Piet Dyrøy, Julius Heitz, Hauke Studier, Sonja Johannsmeier, Tammo Ripken

**Affiliations:** a Leibniz Universität Hannover, Institute of Quantum Optics, Hannover, Germany; b Laser Zentrum Hannover e.V, Life Sciences Department, Hannover, Germany; c Becker & Hickl GmbH, Berlin, Germany

**Keywords:** fluorescence lifetime imaging microscopy, bacterial classification, label-free imaging, NAD(P)H, FAD, metabolic imaging

## Abstract

**Significance:**

Bacterial contamination poses significant risks, particularly in medical settings, where timely identification is crucial for effective treatment. Traditional diagnostic methods often fail to provide rapid results, underlining the need for alternative approaches.

**Aim:**

We investigate the potential of fluorescence lifetime imaging microscopy (FLIM) for fast label-free classification of bacterial species based on their growth states and intrinsic fluorescence characteristics.

**Approach:**

Utilizing two-photon excitation microscopy, we examined the fluorescence lifetimes of the endogenous fluorophores NAD(P)H and FAD in *Escherichia coli* K12, *Staphylococcus aureus*, and *Pseudomonas fluorescens*. Cells were studied in different growth states—exponential, cooled, and dead—and measurements were taken at excitation wavelengths of 740 and 900 nm. The FLIM data were processed using a multi-component exponential decay model and visualized using phasor plots and kernel density estimation.

**Results:**

Results revealed distinct fluorescence lifetime profiles for each species, with clear differences observed in the exponential growth phase. Notably, *E. coli* K12 and *S. aureus* could be distinguished in mixed samples using a single excitation wavelength and emission channel.

**Conclusion:**

These findings demonstrate that FLIM-based metabolic imaging can distinguish between bacterial species without labels, providing a promising basis for rapid, high-resolution diagnostic tools in clinical and research settings. We underscore the potential of FLIM as a powerful tool for bacterial classification, offering advantages in speed and spatial resolution over traditional methods.

## 1 Introduction

Bacterial contamination poses significant risks and complications, particularly in medical settings.^1^^,^^2^ Traditional diagnostic methods, such as biochemical procedures and genetic analyses, may not provide conclusive results in time for the treatment of emergencies, as they often take hours or days and/or require a large number of bacteria. Culture-based identification such aschromogenic culture takes at least 24 h. Polymerase chain reaction (PCR) can deliver results after a few hours, and mass spectrometry (MALDI-TOF) can be performed in less than 30 min after sample preparation, but it is costly and requires a high concentration of bacteria.^3^ Furthermore, these methods do not allow physicians or researchers to assess the native composition of biofilms or identify contaminated niches. To meet this need for fast classification of bacteria in their native state, several alternative image-based methods have been proposed in the past. Hyperspectral imaging or Raman spectroscopy, for instance, were used to successfully identify bacteria at the species level.^4^^,^^5^ However, these methods require specialized expertise and computing power to acquire and interpret the data, and they provide a limited depth of detail and resolution. Other optical methods such as infrared spectroscopy do not preserve the spatial integrity of the samples either. Advantages and disadvantages of established optical methods for identification of bacteria are reviewed elsewhere (e.g., McGoverin et al.).^6^ A viable but less discussed alternative may be found in fluorescence imaging. Andric et al.^7^ showed that two-photon excited fluorescence (TPEF) measurements can distinguish bacterial species based on their respective fluorescence intensities within fixed biofilms.

More information can be gained if the molecular state is included in the fluorescence measurements. Fluorescence lifetime imaging microscopy (FLIM) is an imaging technique based on measuring the decay time of the fluorescence of molecules. Beyond the pure intensity, the analysis of the fluorescence lifetime provides an additional level of information. As endogenous fluorophores, such as the coenzymes NAD(P)H and FAD, can be used for this purpose, it is a label-free imaging method that does not require any additives. This technique can extract the functional state of living cells by identifying whether the coenzymes are present in free or bound states. As the free-to-bound ratio mirrors the metabolic activity of the cell, FLIM is also referred to as metabolic imaging. This approach proved to be particularly useful in cancer research, where metabolic changes are a key feature.^8^^–^^11^Although not fully established in the field of microbiology, individual publications have demonstrated the value of FLIM for investigating bacteria. Most publications focus on the investigation of bacterial metabolism or the visualization of markers.^12^^–^^15^ Bhattacharjee et al.^16^ showed that phasor plots obtained via FLIM can be used to classify bacteria based on their growth states. Sundaramoorthy et al.^17^ successfully used autofluorescence lifetime parameters to distinguish between species in controlled laboratory settings. However, a systematic comparison and classification of different species using FLIM that adequately considers the influence of various growth states has not been published yet. Such a comparison is crucial to fully judge the potential of FLIM for fast species differentiation at high spatial resolution in research and clinical settings.

In this study, we investigate and compare the fluorescence lifetime of intrinsic molecules from three bacterial species: *Escherichia coli* K12 (*E. coli* K12), *Staphylococcus aureus* (*S. aureus*), and *Pseudomonas fluorescens* (*P. fluorescens*). We demonstrate distinct characteristics and their changes with slowing metabolism. Furthermore, we show that *E. coli* K12 can be successfully distinguished from *S. aureus* when present in the same sample, using only one excitation wavelength and emission channel.

## 2 Methods

### 2.1 Sample Preparation

To investigate general fluorescence behavior and its diagnostic relevance, three bacterial species were selected for measurement: *Escherichia coli* K12, *Pseudomonas fluorescens*, and *Staphylococcus aureus*. *Escherichia coli* K12 was chosen as a surrogate for the clinically relevant *Escherichia coli* strains. *Pseudomonas fluorescens* served as a surrogate for *Pseudomonas aeruginosa*, a well-known hospital germ often carrying antibiotic resistance.^1^ *Pseudomonas fluorescens* also plays a minor role in clinical settings.^18^ *Staphylococcus aureus* is a ubiquitous and facultatively pathogenic bacterium.^19^ The three species (one gram-positive, two gram-negative) were chosen to represent a relevant variety of common bacteria connected to clinical routine, without overloading the investigations. Before use, all species were cultured in liquid lysogenic broth (LB) at 37°C and shaken at 100 rpm until they reached their exponential growth phase, which is characterized by high metabolic activity. Three samples were taken from each species at this phase. One was taken as is, one cooled to 5°C to slow down metabolic processes. The third one was heat-treated in an oven at 55°C for 5 min, effectively killing the bacteria. This sample served as a dead reference. In the end, 7  μl of each sample were pipetted onto a microscopy slide and secured with a cover slip for imaging. This whole process was repeated at least 5 times per species to collect independent measurements for each growth state.

### 2.2 Imaging

**Fig. 1:**
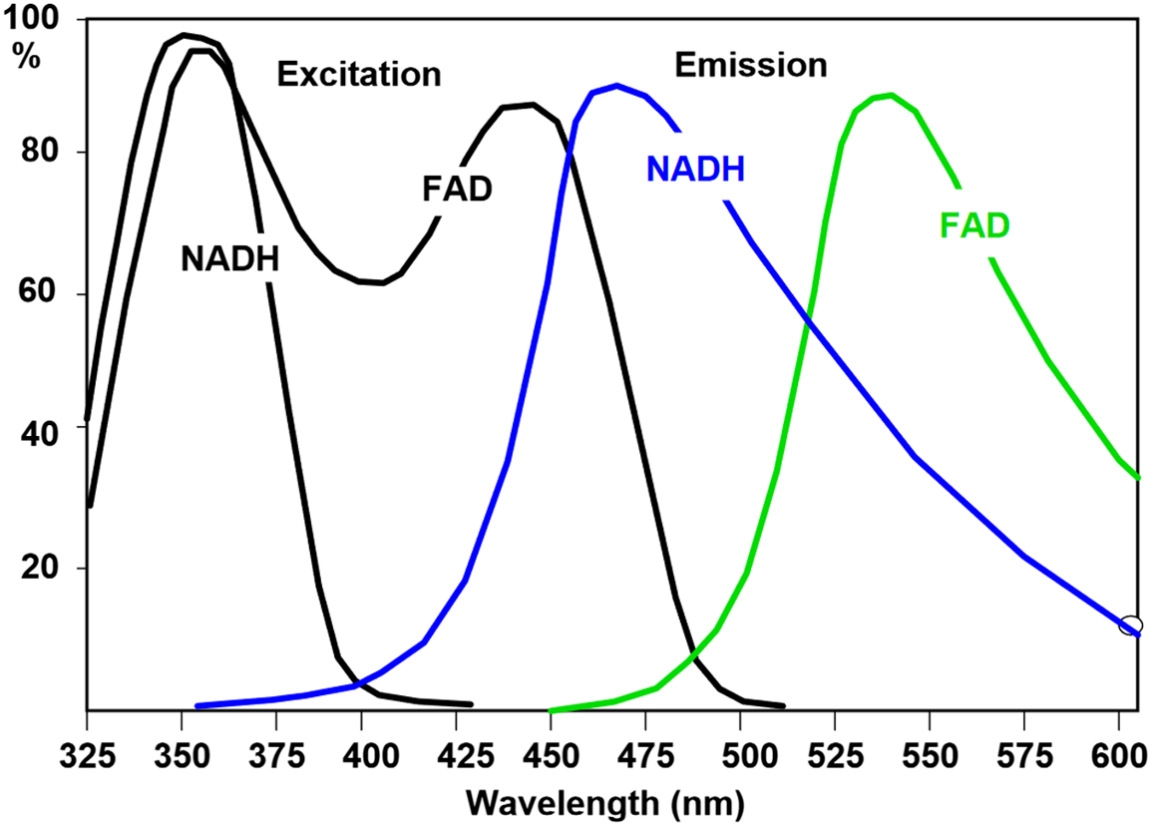
Excitation and emission spectra of NAD(P)H and FAD.^20^ [p. 524, fig. 735].

The FLIM measurements were performed with a modified TrimScope II (LaVision BioTec GmbH—Miltenyi Biotec Company, Germany), using a Chameleon Ultra II (Coherent, USA) laser for excitation. The two-photon microscopy setup was upgraded to a FLIM system with two single-photon detectors HPM-100-40 and two high precision time-triggers SPC-180NX (Becker & Hickl GmbH, Germany) and the corresponding data acquisition suite. This setup was then used to perform the FLIM measurements on the prepared bacteria. NAD(P)H and FAD both exist in bound and unbound (free) states. Unbound NAD(P)H has a fluorescence lifetime of 0.3 to 0.4 ns, whereas bound NAD(P)H has a lifetime of 1.9 to 5.7 ns. Bound and unbound FAD possess lifetimes of 0.003 to 4.55 ns and 2.3 to 2.9 ns, respectively.^9^ The excitation maximum of NAD(P)H lies at 350 nm, with excitable wavelengths ranging from 330 to 370 nm. FAD displays two maxima (350 and 450 nm) and can be excited between 330 and 470 nm (as seen in Fig. 1).^9^^,^^20^^–^^26^ Excitation was performed at 370 and 450 nm. The laser was set to 740 and 900 nm, respectively, to achieve the corresponding two-photon excitation. To filter the emission spectrum, a dichroic mirror split the signal at 495 nm. Two bandpass filters were used to detect the signal: 460/50 nm (Chroma Technology Corp., USA) for the NAD(P)H channel, and 578/105nm (Chroma Technology Corp., USA) for the FAD channel. At 370 nm, both NAD(P)H and FAD are excited. The NAD(P)H signal is clearly distinguishable, but the emission spectrum of NAD(P)H also bleeds into the FAD channel, resulting in a mixed signal in the FAD channel (see Fig. 1).^20^^,^^27^ Sole FAD 2P-excitation was ensured at 450 nm. Here, the NAD(P)H channel was expected to remain empty, and the FAD channel should show a clear signal. Measurement settings include a galvanometric scanner with a 0.18 Hz frame scan rate with size 1020×1020  px. The laser is an 80 MHz pulsed excitation source, which limits the time between two pulses to 12.5 ns. To achieve high temporal resolution, this interval was divided into 1024 equal time bins. As the autofluorescence signal was weak and phototoxicity is to be reduced as much as possible, a laser power of 18 to 20 mW was used, resulting in an acquisition time of 1800 s to reach lifetime photon counts of the bacteria of at least 100 counts at the fluorescence lifetime peak. These measurements always reached at least 10,000 photons per pixel, but not more than 100,000, to ensure reliable multi-component exponential fits.

### 2.3 Image Processing

**Fig. 2:**
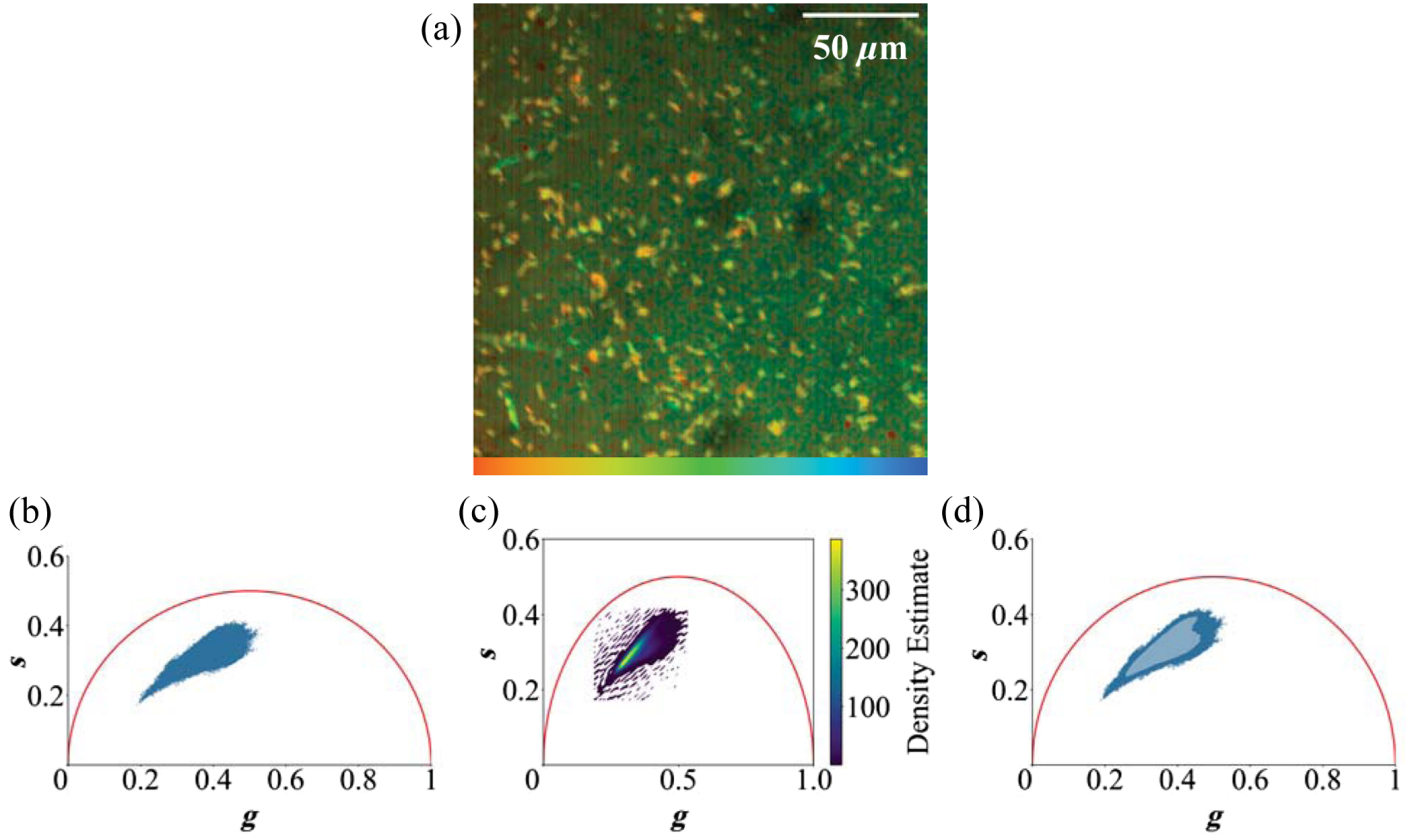
(a) False color image of *E. coli* K12 from the NAD(P)H channel of *E. coli* K12, excited at 740 nm. Red stands for a shorter weighted average lifetime and changes to blue, which indicates a longer weighted average lifetime (1000 to 1900 ps). The corresponding phasor plot (b) and the heat map of the KDE of the phasor plot in panel (c). Panel (d) uses the combined visualization of the KDE of the 99% densest points and a scatter plot of the remaining 1%.

The FLIM data were processed using SPCImage NG software (Becker & Hickl, Berlin, Germany) with a multi-component exponential decay model consisting of three components, assuming the free and bound state of the fluorophore, and a third component containing background noise. To enhance the signal-to-noise ratio, 5-pixel square-binning was applied. Discarding empty pixels, a peak thresholding value of 5 pixels was used. This includes background pixels. Assuming an incomplete multi exponential decay function, a maximum likelihood estimation method was employed to accurately model the decay curves in 10 iterations. This approach allowed for robust fitting of the data. To visualize the complex fluorescence dynamics in each individual pixel, phasor plots were utilized, representing the data using polar coordinates.^28^^,^^29^ Phasor plots compress the complex lifetime information in a visually more intuitive way by reducing the number of factors from 6 (3 lifetimes and 3 amplitude components) to 2. A single decay function will always yield coordinates on the edge of a semicircle, with shorter lifetimes located on the right and longer lifetimes on the left side. In cases of multiple exponential lifetimes, the phasor approach represents the measured decay as a weighted average of the component phasors, resulting in a position lying between the edge points corresponding to the pure lifetimes.^30^ An exemplary measurement as intensity image is shown in Fig. 2(a) with the corresponding phasor plot in Fig. 2(b). The recorded data were the basis for distinguishing the imaged bacteria. To include all available information, classification was performed using the raw data, visualized as phasor plots thathighlight unique characteristics of each species’ autofluorescence signal. Data were collected from multiple measurements at 740 and 900 nm, each containing multiple individual bacterial cells. To facilitate comparison between samples within the same plot, a kernel density estimation (KDE) was used to visualize this data and to effectively display complex distributions without overlapping points. A heat map of the KDE phasor plot for *E. coli* K12’s NAD(P)H channel at 740 nm is shown in Fig. 2(c). By plotting the densest 99% of data points as a contour area and the remaining 1% as a scatterplot, we preserved both overall trends of the bacteria signal and the background and unique characteristics [Fig. 2(d)]. This allows for an intuitive view of the shape of the phasor plots without overplotting the densest part, but including the outliers, which may help distinguish the phasor plot clouds.

### 2.4 Statistical Analysis

All analyses used stratified sampling with equal draws per measurement file (seed 42), with caps of 50,000 data points per group for pairwise distances and 10,000 per group for bootstrap confidence intervals. These choices control computation while maintaining comparable representation across measurements. Distributional differences were quantified with the Jensen–Shannon distance (JSD, base 2) computed on 2D histograms. A pooled fixed support was used with Freedman–Diaconis bin counts per axis, with a small constant $\epsilon =10^{-10}$ added to avoid zeros while retaining shared binning across comparisons. KDE bandwidths were selected by cross-validation. JSD point estimates were computed using fixed pooled histogram edges, and sensitivity checks verified conclusions under ±25% perturbations of bin widths.^31^^,^^32^ Geometric separation was summarized by a combined Wasserstein transport $W_{\text{comb}}=\sqrt{W(g{)}^{2}+W(s{)}^{2}}$, where W(g) and W(s) are 1D Wasserstein distances along g and s, respectively, in native phasor units.^33^ Centroid separation in the joint (g,s) space was measured with the Mahalanobis distance $D_{M}=\sqrt{\left(\mu_{1}-\mu_{2}{)}^{⊺}S_{p}^{-1}(\mu_{1}-\mu_{2}\right)}$. A pooled Ledoit–Wolf shrinkage covariance was used to stabilize estimation under unequal dispersions. Uncertainty for pairwise metrics used 95% bootstrap confidence intervals (CI, percentile and bias-corrected with a=0) with B=500 resamples per pair, resampling occurs with replacement within groups at the pixel level. Point estimates were taken from the non-bootstrapped stratified sets. Bootstrap CIs are reported for W(g), W(s), $W_{\text{comb}}$, and $D_{M}$. JSD is presented as a point estimate using fixed pooled supports.

## 3 Results

### 3.1 Comparison of Fluorescence Lifetimes within a Single Species

**Fig. 3:**
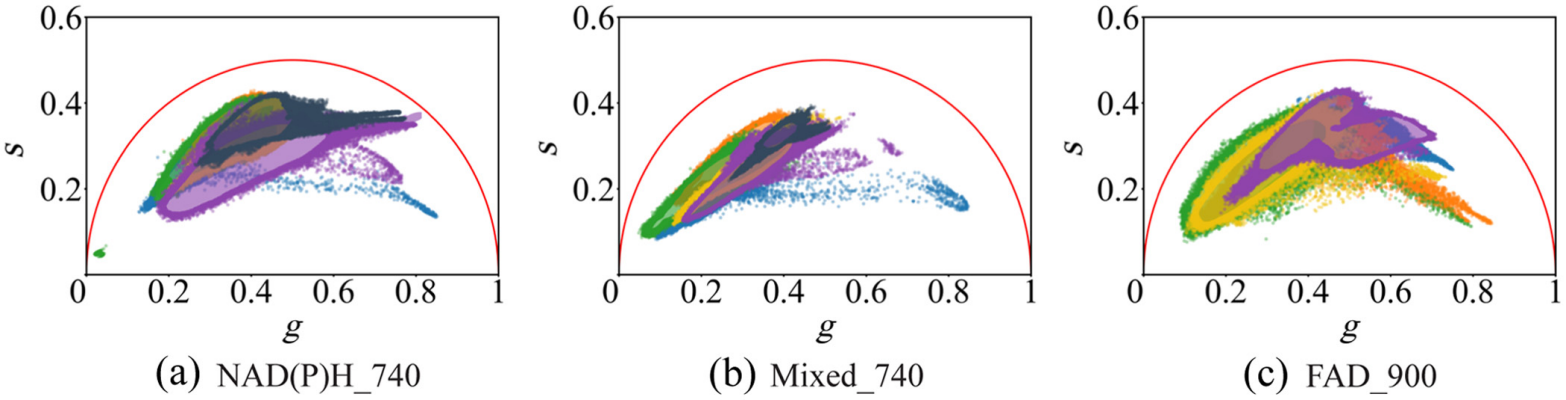
Phasor plot from *E. coli* K12 excited at 740 and 900 nm. Each color represents one sample. Panel (a) used a 460/50 nm filter for NAD(P)H, whereas (b) and (c) used a 578/105nm filter for FAD. Visualized and partly grouped using a KDE for visualization.

To gain insight into the measurements, we examined all four image channels, corresponding to all combinations of excitation wavelengths and detection channels. We present *E. coli* K12 as a representative example in this section. The same analysis was performed for all three bacterial species and all growth states. Figure 3 presents phasor plots for *E. coli* K12 in the exponential growth phase. The plots are divided into three channels: (a) corresponds to the NAD(P)H emission channel excited at 740 nm, whereas(b) and (c) correspond to the FAD emission channel, excited at 740 and 900 nm, respectively. For easier reading, the channels will be referred to as NAD(P)H_740, Mixed_740, FAD_900. Note that the NAD(P)H channel is absent in the 900 nm excitation plot, as no fluorophore is addressed by this combination of excitation and emission wavelengths (see Fig. 1). Thus, no signal was detected. At least five samples per species and metabolic state were measured under the same conditions to assess the reproducibility of the phasor plot’s shape and range. The results revealed a high degree of overlap, but also some variability within each channel. The measurements in (a) and (b) were recorded simultaneously and thus were obtained from the same cells. The subsequent measurement shown in (c) was performed at a different location on the sample to exclude any influence of the first irradiation on the fluorescence lifetimes. It can also be seen that a small number of pixels deviate from the densest part of the decay times, potentially holding important information.

### 3.2 Comparison between Species

**Fig. 4:**
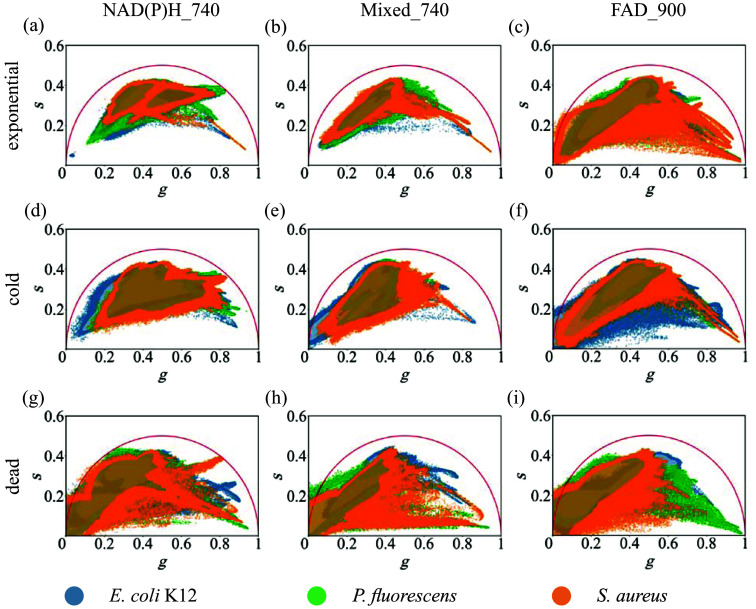
Overview of phasor plots of *E. coli* K12 (blue), *P. fluorescens* (green), and *S. aureus* (orange). The top row contains the measurements in the exponential growth phase, the middle row the cold bacteria, and the bottom row the dead bacteria.

After initial assessment and removal of outliers, all data obtained for one species were pooled and summarized with KDE to enable direct comparison across species and conditions. Figure 4 is organized by growth state in rows (exponential, cold, dead) and by imaging channel in columns (NAD(P)H_740, Mixed_740, FAD_900). Summaries of pairwise statistical analyses are reported as triplets (exponential/cold/dead) in Table 1. The metrics were chosen to quantify separability of the point clouds without assuming a particular distribution for the population. In the exponential state, pooled distributions overlap, yet they show a consistent species-dependent structure. Mixed_740 exhibits the largest separations across pairs (JSD 0.59 to 0.76, $W_{\text{comb}}\;0.03\text{–}0.09$, and $D_{M}\;0.91\text{–}1.84$), where NAD(P)H_740 shows moderate (JSD 0.61 to 0.65, $W_{\text{comb}}\;0.03\text{–}0.06$, and $D_{M}\;0.52\text{–}0.94$), and FAD_900 contains the least separation (JSD 0.33 to 0.51, $W_{\text{comb}}\;0.01\text{–}0.05$, and $D_{M}\;0.02\text{–}0.15$). Multimodality for *S. aureus* near g≈0.3 and g≈0.5 is visible in Fig. 4(a). Under cooling, Mixed_740 maintains detectable separations, but the strength is generally reduced relative to exponential (JSD 0.61–0.74, $W_{\text{comb}}\;0.02\text{–}0.10$, and $D_{M}\;0.27\text{–}1.03$), with a modest increase for *E. coli* K12 versus *P. fluorescens*. NAD(P)H_740 remains at a modest level (JSD 0.52–0.63, $W_{\text{comb}}\;0.02\text{–}0.07$, and $D_{M}\;0.28\text{–}0.71$). FAD_900 increases markedly versus exponential (JSD 0.46–0.67, $W_{\text{comb}}\;0.04\text{–}0.10$, and $D_{M}\;0.71\text{–}1.04$). Mixed_740_cold shows two density regions for *P. fluorescens* around s≈0.35 and s≈0.2 [Fig. 4(e)]. In the dead state, distributions broaden across channels, increasing pixel-level overlap but amplifying population-level separations in specific channels. Mixed_740 shows the largest magnitudes (JSD 0.63–0.90, $W_{\text{comb}}\;0.04\text{–}0.28$, and $D_{M}\;0.91\text{–}3.11$), with the largest values observed in contrasts involving *E. coli* K12. FAD_900 is intermediate (JSD 0.55–0.85, $W_{\text{comb}}\;0.07\text{–}0.21$, and $D_{M}\;0.54\text{–}1.66$), whereas NAD(P)H_740 remains smaller but detectable (JSD 0.44–0.82, $W_{\text{comb}}\;0.02\text{–}0.14$, and $D_{M}\;0.14\text{–}1.19$). Overall, separability follows a channel- and state-dependent hierarchy: Separation in Mixed_740 is highest for exponential and dead, values for FAD_900 increase under cooling and are intermediate in dead, and separation in NAD(P)H_740 is consistently smaller but present. Point estimates are shown in Table 1 and bootstrap confidence intervals for W(g), W(s), $W_{\text{comb}}$, and $D_{M}$ are provided in Table S1 in the Supplementary Material.

**Table 1:** Pairwise separations across states shown as triplets (exp/cold/dead). eck12 signifies *E. coli* K12, sa for *S. aureus*, and pf for *P. fluorescens*. JSD summarizes distributional differences, $W_{\text{comb}}=\sqrt{W(g{)}^{2}+W(s{)}^{2}}$ summarizes axis-wise transport, and $D_{M}$ is the Mahalanobis distance in joint (g,s) space using a pooled Ledoit–Wolf covariance.

Channel	Pair	JSD (exp/cold/dead)	$W_{\text{comb}}$ (exp/cold/dead)	$D_{M}$ (exp/cold/dead)
NAD(P)H_740	eck12–pf	0.61/0.63/0.72	0.06/0.06/0.12	0.63/0.55/1.13
	eck12–sa	0.64/0.61/0.82	0.04/0.07/0.14	0.94/0.71/1.19
	pf–sa	0.65/0.52/0.44	0.03/0.02/0.02	0.52/0.28/0.14
Mixed_740	eck12–pf	0.67/0.73/0.90	0.07/0.09/0.28	1.01/1.03/3.11
	eck12–sa	0.76/0.74/0.87	0.09/0.10/0.27	1.84/0.97/2.74
	pf–sa	0.59/0.61/0.63	0.03/0.02/0.04	0.91/0.27/0.91
FAD_900	eck12–pf	0.33/0.67/0.84	0.01/0.10/0.18	0.02/1.04/1.32
	eck12–sa	0.51/0.46/0.85	0.05/0.09/0.21	0.14/0.71/1.66
	pf–sa	0.48/0.59/0.55	0.05/0.04/0.07	0.15/0.71/0.54

### 3.3 Distinction between Two Species

**Fig. 5:**
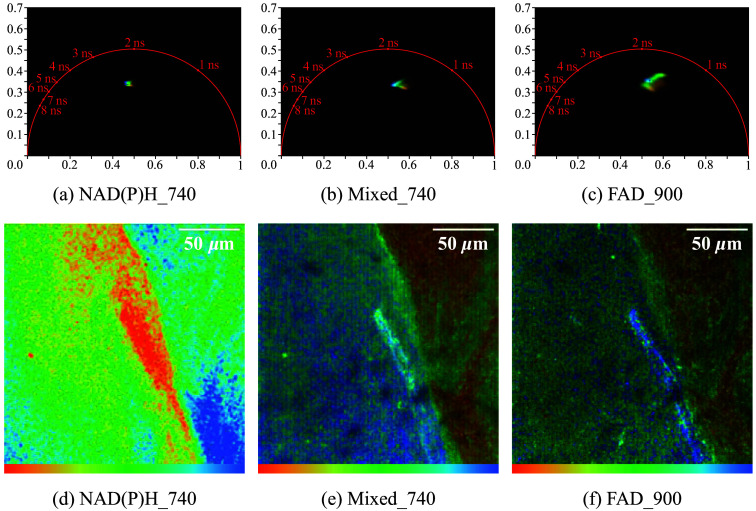
Phasor plots with KDE of a combined measurement of *E. coli* K12 (left) and *S. aureus* (right). (a), (b), and (c) show the respective phasor plots and (d) shows the image for (b) in false color representation. The pixels in the phasor plots are linked to the corresponding image. Red stands for a shorter weighted average lifetime and changes to blue, which indicates a longer weighted average lifetime. All channels show a difference in the average lifetime between the bacterial species. The clearest difference can be seen in the combined signal in (b).

In an additional setting, *E. coli* K12 and *S. aureus* were taken directly from LB agar plates and spread out on a microscope slide. The species were not mixed, but spread directly adjacent to each other, with *E. coli* K12 to the left and *S. aureus* to the right. FLIM imaging was performed at the boundary as described before. For this measurement, a lower laser power of around 10 mW to 12 mW and an acquisition time of 900 s was used, as the signal was much stronger. This approach allowed us to identify any distinct signals that would assign any individual cell to either of the two species. Moreover, the bacteria interact with each other, allowing us to examine the interface between them. Figure 5 presents both the images and phasor plots of all three relevant channels. The KDE representation was not used here. The lifetimes in the phasor plot are more densely packed and exhibit fewer extreme values. The representations were directly obtained from the evaluation software by Becker & Hickl and are displayed in false colors, where red corresponds to a short lifetime and blue to a long one. However, only weighted mean lifetimes are considered here. Individual components are not examined separately. This color representation is consistent between the phasor plots and their corresponding images. The depicted area corresponds to $196\;{\mu m}^{2}$. In the NAD(P)H channel [Fig. 5(d)], it is apparent that the boundary region of *E. coli* K12 exhibits a shorter lifetime compared withthe cells located further toward the sides. A region with longer lifetimes is visible in the bottom right corner, but otherwise, the lifetimes of both bacteria are similar. We observe a lifetime range of 1079 to 1283 ps. Upon examining the FAD channel [Fig. 5(c)], we also observe a considerably smaller range of lifetimes, although it is still larger than that observed in Fig. 5(a). In the FLIM image, which is colored according to lifetimes between 507 and 960 ps, we can see that *S. aureus* tends to exhibit shorter lifetimes. By contrast, *E. coli* K12 displays a more mixed pattern, with the interface exhibiting longer lifetimes and the left region being highly heterogeneous, featuring areas with both very short and very long lifetimes. Finally, examining the FAD channel excited at 740 nm [Fig. 5(e)], we observe that the weighted mean lifetime falls between those of the two pure channels (753 to1097 ps). A pronounced distinction between *E. coli* K12 and *S. aureus* is visible in this channel. All measurements show much less variability in the weighted phasor than the bacteria in the liquid medium.

## 4 Discussion

In this study, we utilized the autofluorescence of NAD(P)H and FAD to investigate the potential of metabolic imaging to create species-specific signatures using FLIM data. All bacterial species exhibit some variability between measurements, as became evident from individual measurements. This was also observed by Bhattacharjee et al.^16^ The bacteria in the FLIM images were not selected or sorted prior to measurement, and therefore, the data still contain background pixels that could influence the results. However, this also reflects a more realistic scenario, as it is rarely possible to work with pure bacterial cultures in real-world environments. This approach will be particularly important in future studies aimed at developing diagnostic tools that can function in complex and diverse environments.

Statistical analyses focussed on descriptive metrics and effect sizes rather than on significance testing. Beyond centroid shifts, species differed in the geometry of their phasor distributions (compactness, elongation/orientation, and modality), which is visible in the KDE overlays. Shape-aware metrics summarize complementary aspects: Jensen–Shannon divergence emphasizes support and modality differences, whereas axis-resolved and combined Wasserstein distances emphasize location and spread in native units, jointly capturing separation even when centroids overlap. Channel- and state-dependent patterns are consistent with the quantitative tables and figures. Under exponential conditions, Mixed_740 provided the strongest separability, where NAD(P)H_740 was moderate, and FAD_900 limited, matching the overlays [Figs. 4(a)–4(c)]. Under cooling, FAD_900 became more informative, Mixed_740 generally decreased relative to exponential (with a modest increase for the*P. fluorescens* contrast), and NAD(P)H_740 remained at a modest level [Fig. 4(f)]. In the dead state, broader distributions increased pixel-level overlap. Nevertheless, population-level separability persisted, with the largest values in Mixed_740, intermediate values in FAD_900, and smaller but detectable values in NAD(P)H_740 [Figs. 4(g)–4(i)]. However, it is not entirely clear whether these changes are still biologically relevant. Pixel-level dependence within images complicates single-pixel assignment and precision, if ignored. Accordingly, performance assessments should operate at the measurement level, and classification should exploit distributional information across channels instead of single-channel centroids. A machine-learning approach integrating multi-channel, distribution-aware features (for example, divergence, transport, and robust covariance summaries) is a promising next step, with uncertainty quantified via bootstrap intervals and evaluation performed at the measurement-file level. Overall, the observed hierarchy is: Mixed_740 strongest (exponential and dead), FAD_900 increasing under cooling, and NAD(P)H_740 consistently modest. This supports multi-channel integration for robust discrimination in realistic, background-rich settings.

This study did not specifically investigate the origin of the fluorescence signals, but rather assumed the measurement of NADPH and FAD based on their excitation/emission spectra. The changes in the signal depending on the metabolic state of the bacteria; however, indicates that metabolic molecules play a significant role in these measurements,^16^^,^^34^^,^^35^ although Sundaramoorthy et al.^17^ also demonstrated the use of general autofluorescence. Our findings support the broader potential of these optical techniques and extend their application to more complex and realistic sample environments, such as swabs and diluted solutions, using samples with an optical density of less than one. The direct comparison of the two species taken from agar plates and spread with high cell density resulted in a much smaller area of pixels in the phasor plot than measurements performed with liquid media. This can likely be attributed to the higher corresponding signal density [Fig. 5(e)]. Although the NADH channel at 740 nm and the FAD channel at 900 nm showed only small differences, the mixed channel allowed for a clear distinction between the two species. The fact that only one channel is needed for successful distinction of *E. coli* K12 versus *S. aureus* holds interesting implications for future research. Especially the NAD(P)H channel at 740 nm showed a shortened mean lifetime in the area of contact between the bacteria, specifically among the *E. coli* K12 cells. Shorter NAD(P)H fluorescence lifetimes indicate a metabolic shift toward glycolysis, which could be caused by metabolic stress.^16^ Although the actual cause of such changes was not investigated in this study, the established protocols can be used to identify the impact of interactions, such as metabolic cross-talk or physical adhesion on the species discriminability via FLIM.

Performing species classification with a single measurement would allow for more reliable results, automated characterization and high spatial resolution. The fluorescence signals measured in the FAD channel with 740 nm excitation possess lifetimes that lie roughly in between those expected for either NAD(P)H or FAD. In this configuration, both NAD(P)H and FAD are excited, and emission from both molecules is detectable, resulting in a mixed signal. The corresponding fit function might then yield lifetime values in between those of the individual molecules. This should, however, be investigated further to quantify the informative value of the measurements in this channel under different circumstances. Cao et al.^27^ proposed the use of only 800 nm for excitation to cut down on measurement time and reduce the presence of NAD(P)H in the FAD channel.Although the signals from the two molecules cannot be cleanly separated this way, our results suggest that reducing the number of channels can be valid in settings where a qualitative rather than quantitative outcome is sought.

In the present setup, the acquisition time of ∼1800  s was relatively long. Several factors contributed to this, including the comparatively slow galvo scanner of the Trimscope II, which resulted in a frame time of 5.56 s, as well as idle periods between consecutive frame clocks. Capturing autofluorescence from bacteria in exponential growth phase within a liquid medium requires a careful balance between signal-to-noise ratio, total measurement time, photodamage, image quality, and laser power. In this study, emphasis was placed on maximizing the signal-to-noise ratio. A second measurement at the same position revealed evidence of light-induced damage, with average lifetimes being shorter in the previously illuminated region compared with untreated areas. The exact reason for this observation could not be identified within our setup, and the available literature lacks investigations of this phenomenon in bacteria, and often does not report the corresponding excitation parameters. Alam et al.^36^ obtained similar measurements after photodamage to HeLa cells, although such observations are not directly transferrable to bacterial cells. To avoid any such excitation artifacts in our study, data were never collected from repeated measurements at the same location.

Also, it is not clear how many photons are needed exactly for the phasor plots. Further research with an optimized system should lead to much shorter measurement times—even more so, if the signal can be obtained by using only one excitation wavelength, as suggested by the results.

## 5 Conclusion

In this paper, we demonstrated that bacterial species can be distinguished via metabolic imaging of NAD(P)H and FAD. This was especially visible in the phasor plots of the mixed channel at 740 nm. The single measurement held enough information to distinguish *E. coli* K12 and *S. aureus*. Parameter ratios, based on bound and unbound NAD(P)H and FAD lifetimes, are used in cancer and stem-cell research, such asthe fluorescence-lifetime redox ratio (FLIRR index) or optical redox ratio (ORR). These ratios provide further insight into the metabolism of the cells and a contrast mechanism fordistinguishing features.^11^^,^^17^^,^^37^ Such ratios might be adaptable to species classification. They were not used in this paper, but will be explored in further research. Future analyses will combine high-level comparisons of relative lifetime shifts across species, metabolic states, and imaging channels with machine-learning models that consider entire phasor distributions, including outliers, to retain rare but diagnostically relevant regimes. This integrated approach is expected to expose the full potential of metabolic imaging for bacterial classification under realistic conditions, leveraging ongoing advances in FLIM data processing and machine learning to make it a powerful tool in microbiology.

## Supplementary Material

10.1117/1.JBO.31.1.016502.s01

## Data Availability

All relevant code, data, and materials are available from the authors upon request from the corresponding author.
