# Supplementary material for: Phasor-based FLIM analysis of NAD(P)H and FAD autofluorescence for label-free bacterial classification
Source: J Biomed Opt. 2026 Jan 17;31(1):016502. doi: 10.1117/1.JBO.31.1.016502 (PMC12811912; doi:10.1117/1.JBO.31.1.016502)
Supplement: Supplementary file 1 [file JBO_031_016502_SD001.pdf]

Table S1: Bootstrap precision for pairwise separations by channel, state, and species pair. eck12 signifies for *E. coli* K12, sa for *S. aureus* and pf for *P. fluorescens*. Entries show the point estimate followed by the 95% percentile bootstrap confidence interval in brackets. Resampling was performed at the measurement-file level (cluster bootstrap).

Channel	State	Pair	$W(g)$ (pt [95% CI])	$W(s)$ (pt [95% CI])	D_M (pt [95% CI])
NAD(P)H_740	exp	eck12–pf	0.0526 [0.0503, 0.0553]	0.0251 [0.0242, 0.0265]	0.6283 [0.6066, 0.6619]
		eck12–sa	0.0296 [0.0287, 0.0313]	0.0330 [0.0322, 0.0341]	0.9404 [0.9121, 0.9747]
		pf–sa	0.0291 [0.0276, 0.0314]	0.0183 [0.0178, 0.0193]	0.5198 [0.4931, 0.5492]
	cold	eck12–pf	0.0506 [0.0488, 0.0536]	0.0236 [0.0226, 0.0255]	0.5510 [0.5285, 0.5873]
		eck12–sa	0.0617 [0.0591, 0.0655]	0.0349 [0.0333, 0.0363]	0.7138 [0.6941, 0.7430]
		pf–sa	0.0196 [0.0165, 0.0204]	0.0111 [0.0097, 0.0122]	0.2779 [0.2320, 0.2854]
	dead	eck12–pf	0.1068 [0.1044, 0.1097]	0.0576 [0.0558, 0.0600]	1.1345 [1.1209, 1.1787]
		eck12–sa	0.1215 [0.1194, 0.1249]	0.0681 [0.0656, 0.0700]	1.1885 [1.1688, 1.2362]
		pf–sa	0.0159 [0.0149, 0.0196]	0.0173 [0.0164, 0.0186]	0.1412 [0.1242, 0.1755]
Mixed_740	exp	eck12–pf	0.0429 [0.0409, 0.0441]	0.0504 [0.0486, 0.0512]	1.0061 [0.9695, 1.0277]
		eck12–sa	0.0503 [0.0489, 0.0515]	0.0773 [0.0754, 0.0783]	1.8390 [1.7635, 1.8674]
		pf–sa	0.0105 [0.0097, 0.0118]	0.0269 [0.0257, 0.0282]	0.9085 [0.8668, 0.9406]
	cold	eck12–pf	0.0715 [0.0700, 0.0738]	0.0574 [0.0558, 0.0598]	1.0283 [0.9904, 1.0477]
		eck12–sa	0.0685 [0.0662, 0.0705]	0.0668 [0.0645, 0.0684]	0.9650 [0.9370, 0.9913]
		pf–sa	0.0145 [0.0138, 0.0155]	0.0117 [0.0115, 0.0135]	0.2740 [0.2365, 0.2956]
	dead	eck12–pf	0.2294 [0.2277, 0.2320]	0.1634 [0.1624, 0.1654]	3.1101 [3.0615, 3.1691]
		eck12–sa	0.2030 [0.2012, 0.2059]	0.1772 [0.1756, 0.1789]	2.7437 [2.7186, 2.8147]
		pf–sa	0.0279 [0.0266, 0.0306]	0.0232 [0.0221, 0.0241]	0.9060 [0.8747, 0.9477]
FAD_900	exp	eck12–pf	0.0115 [0.0103, 0.0138]	0.0064 [0.0063, 0.0078]	0.0228 [0.0073, 0.0573]
		eck12–sa	0.0448 [0.0425, 0.0452]	0.0308 [0.0293, 0.0314]	0.1433 [0.1309, 0.1870]
		pf–sa	0.0403 [0.0392, 0.0423]	0.0318 [0.0315, 0.0335]	0.1517 [0.1203, 0.1781]
	cold	eck12–pf	0.0675 [0.0644, 0.0689]	0.0680 [0.0654, 0.0694]	1.0396 [0.9989, 1.0564]
		eck12–sa	0.0741 [0.0720, 0.0786]	0.0558 [0.0543, 0.0590]	0.7066 [0.6809, 0.7370]
		pf–sa	0.0386 [0.0377, 0.0402]	0.0177 [0.0175, 0.0191]	0.7130 [0.6895, 0.7548]
	dead	eck12–pf	0.1667 [0.1630, 0.1678]	0.0585 [0.0573, 0.0594]	1.3239 [1.2709, 1.3764]
		eck12–sa	0.1999 [0.1960, 0.2048]	0.0763 [0.0736, 0.0794]	1.6639 [1.6205, 1.7099]
		pf–sa	0.0570 [0.0541, 0.0604]	0.0485 [0.0457, 0.0489]	0.5367 [0.5146, 0.5711]
